# Fucosylated Chondroitin Sulfates from the Sea Cucumbers *Paracaudina chilensis* and *Holothuria hilla*: Structures and Anticoagulant Activity

**DOI:** 10.3390/md18110540

**Published:** 2020-10-28

**Authors:** Nadezhda E. Ustyuzhanina, Maria I. Bilan, Andrey S. Dmitrenok, Alexandra S. Silchenko, Boris B. Grebnev, Valentin A. Stonik, Nikolay E. Nifantiev, Anatolii I. Usov

**Affiliations:** 1 N.D. Zelinsky Institute of Organic Chemistry, Russian Academy of Sciences, Leninsky Prospect 47, 119991 Moscow, Russia; bilan@ioc.ac.ru (M.I.B.); dmt@ioc.ac.ru (A.S.D.); nen@ioc.ac.ru (N.E.N.); 2 G.B. Elyakov Pacific Institute of Bioorganic Chemistry, Far Eastern Branch of the Russian Academy of Sciences, Prospect 100 let Vladivostoku 159, 690022 Vladivostok, Russia; sialexandra@mail.ru (A.S.S.); grebnev_bor@mail.ru (B.B.G.); stonik@piboc.dvo.ru (V.A.S.)

**Keywords:** sea cucumber, *Holothuria hilla*, *Paracaudina chilensis*, fucosylated chondroitin sulfate, anticoagulant activity

## Abstract

Fucosylated chondroitin sulfates (FCSs) **PC** and **HH** were isolated from the sea cucumbers *Paracaudina chilensis* and *Holothuria hilla*, respectively. The purification of the polysaccharides was carried out by anion-exchange chromatography on a DEAE-Sephacel column. The structural characterization of the polysaccharides was performed in terms of monosaccharide and sulfate content, as well as using a series of nondestructive NMR spectroscopic methods. Both polysaccharides were shown to contain a chondroitin core [→3)-β-d-GalNAc (N-acethyl galactosamine)-(1→4)-β-d-GlcA (glucuronic acid)-(1→]_n_, bearing sulfated fucosyl branches at O-3 of every GlcA residue in the chain. These fucosyl residues were different in their pattern of sulfation: **PC** contained Fuc2*S*4*S* and Fuc4*S* in a ratio of 2:1, whereas **HH** included Fuc2*S*4*S*, Fuc3*S*4*S*, and Fuc4*S* in a ratio of 1.5:1:1. Moreover, some GalNAc residues in **HH** were found to contain an unusual disaccharide branch Fuc4*S*-(1→2)-Fuc3*S*4*S*-(1→ at O-6. Sulfated GalNAc4*S*6*S* and GalNAc4*S* units were found in a ratio of 3:2 in **PC** and 2:1 in **HH**. Both polysaccharides demonstrated significant anticoagulant activity in a clotting time assay, which is connected with the ability of these FCSs to potentiate the inhibition of thrombin and factor Xa in the presence of anti-thrombin III (ATIII) and with the direct inhibition of thrombin in the absence of any cofactors.

## 1. Introduction

Fucosylated chondroitin sulfates (FCSs) are the unique polysaccharides found exclusively in the body walls of sea cucumbers. These biopolymers are composed of d-glucuronic acid, *N*-acetyl-d-galactosamine, l-fucose, and sulfate residues [1,2,3]. A chondroitin core of FCSs [→3)-β-d-GalNAc-(1→4)-β-d-GlcA-(1→]_n_ contains α-l-fucosyl branches attached to O-3 of GlcA or to O-6 of GalNAc [1,2,3,4,5,6]. Sulfate groups may occupy different positions of GalNAc, Fuc, and even GlcA residues. Depending on the species of sea cucumber, FCSs include GalNAc units sulfated at O-4 or at both O-4 and O-6; fucosyl branches Fuc2*S*4*S*, Fuc3*S*4*S*, and Fuc4*S*; as well as GlcA residues sulfated at O-3 or at both O-2 and O-3 [3,4,5,6,7,8]. In addition, difucosyl branches attached to O-3 of GlcA are known as very rare structural fragments of FCSs. Thus, FCS from *Holothuria* (*Ludwigothuria*) *grisea* was shown to contain the branch α-l-Fuc-(1→2)-α-l-Fuc3*S*-1→ [9]. The branch α-l-Fuc-(1→2)-α-l-Fuc3*S*4*S*-1→ was observed in FCS from *Eupentacta fraudatrix* [8]. FCS from *Holothuria lentiginosa* contains the fragment α-l-Fuc-(1→3)-α-l-Fuc4*S*-1→ linked also to O-3 of GlcA [10].

The fine structure of FCSs significantly influences biological activity, which is connected with the interaction of polysaccharides with different proteins. Thus, the intensively studied anticoagulant activity of FCSs was shown to be determined by their ability to potentiate the inhibition of thrombin and factor Xa in the presence of anti-thrombin III (ATIII) [1,3,5,6,11,12,13,14]. Other mechanisms mediated by heparin cofactor II and FXase were also considered [15,16,17]. The presence of fucosyl branches sulfated at O-4 in FCSs was found to be essential for the anticoagulant effect [13,18]. The anti-inflammatory activity of FCSs is connected with their binding to P- and L-selectins [19]. Recently, the anti-angiogenic effect of FCS from *Hemioedema spectabilis* was demonstrated in vitro [20], which is probably mediated by the interaction of the polysaccharide with growth factors [21].

In this communication, we describe the structural characterization of two FCSs (**PC** and **HH**) isolated from the sea cucumbers *Paracaudina chilensis* and *Holothuria hilla*, respectively. The anticoagulant activity of these polysaccharides was studied in vitro.

The sea cucumber *Holothuria hilla*, belonging to the order Holothuriida, is a widely distributed species in the South China Sea, especially near the shore of Fujian Province of China, Dongshan Island, and the Vietnamese seashore. The chemical composition of this sea cucumber was studied in 2006–2007 by Chinese researchers, who isolated and elucidated the structures of three novel triterpene glycosides, hillasides A–C [22,23], together with the previously known holothurins A and B. These glycosides are xylosides of holostane aglycones, differing in their structures of aglycones and carbohydrate side chains. It is interesting to mention that one of them, hillaside C, contained a disaccharide chain composed of two xylose residues, which was found in the triterpene glycosides of sea cucumbers for the first time. Hillasides were shown to exhibit significant cytotoxic activity (in vitro). The polysaccharides of *H. hilla* have not been previously studied.

*Paracaudina chilensis* belongs to the order Molpadiida, the representatives of which have been poorly studied in regard to their chemistry. A new triterpene glycoside, caudinoside A, was isolated from this sea cucumber (named *Paracaudina ransonetii*) in 1986. The structure of native aglycone, 3β-hydroxy-16-ketoholosta-9(11),25-diene, and the monosaccharide composition of the carbohydrate chain (xylose, quinovose, glucose, and 3-O-methylglucose in the ratio of 1:1:3:1) were established, but the whole chemical structure of caudinoside A remains unelucidated [24]. In addition, the amino acid sequence of the major globin isolated from coelomic cells of *P. chilensis* was determined [25], and gelatin hydrolysates were shown to possess antioxidant activity, demonstrating a reasonable radical scavenging effect and preventing the damage of rabbit liver and mitochondria (the species name was erroneously written as ‘chinens’ in this publication) [26]. As in the case of *H. hilla*, the polysaccharide composition of *P. chilensis* has not been investigated previously.

## 2. Results and Discussion

Crude extracts of sulfated polysaccharides were obtained from the body walls of sea cucumbers *Paracaudina chilensis* and *Holothuria hilla* by the conventional solubilization of biomass in the presence of papain [27] followed by the treatment of the extract with hexadecyl-trimethylammonium bromide to precipitate the sulfated components, which were then transformed into water-soluble sodium salts by stirring the components with NaI in ethanol. According to their composition, crude preparations contained sulfated fucans and FCS as the main components. Both crude extracts were subjected to anion-exchange chromatography on a DEAE-Sephacel column. The fractions eluted with 1.0 M NaCl were designated as **PC** for *P. chilensis* and **HH** for *H. hilla*. These preparations contained GlcA, GalNAc, Fuc, and sulfate in ratios (Table 1) typical for holothurian FCSs. The detection of minor Gal and GlcN in hydrolysates was explained by the possible presence of small amounts of other glycosaminoglycans, which could not be eliminated by anion-exchange chromatography. The molecular weights of the polysaccharides were estimated as 28.9 kDA for **PC** and 26.7 kDa for **HH** by gel-permeation chromatography [28].

**Figure 1:**
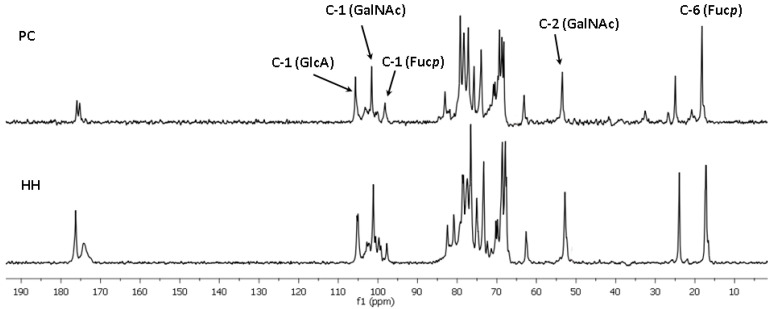
The ^13^C NMR spectra of the fucosylated chondroitin sulfates **PC** and **HH**.

**Figure 2:**
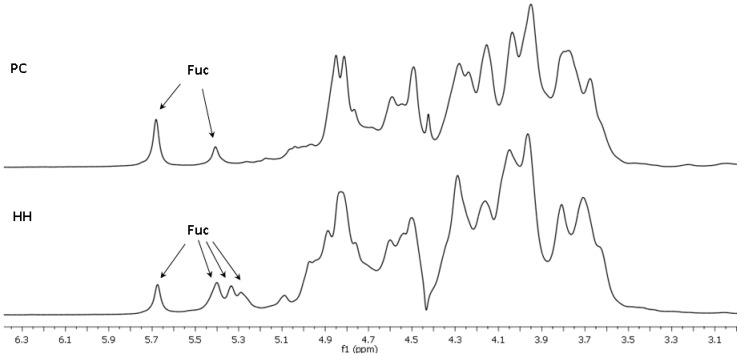
Fragments of ^1^H NMR spectra of the fucosylated chondroitin sulfates **PC** and **HH**.

The structures of polysaccharides **PC** and **HH** were characterized in more detail using NMR spectroscopic methods. The presence of Fuc, GalNAc, and GlcA units in both polysaccharides was confirmed by the characteristic values of chemical shifts of C-6 for Fuc (δ 17.2 ppm) and GlcA (δ 176.0 ppm), as well as of C-2 for GalNAc (δ 52.7 ppm) in ^13^C NMR spectra (Figure 1). The anomeric regions in the ^1^H NMR spectra of the polysaccharides were quite different, indicating the presence of different fucosyl branches (Figure 2).

**Figure 3:**
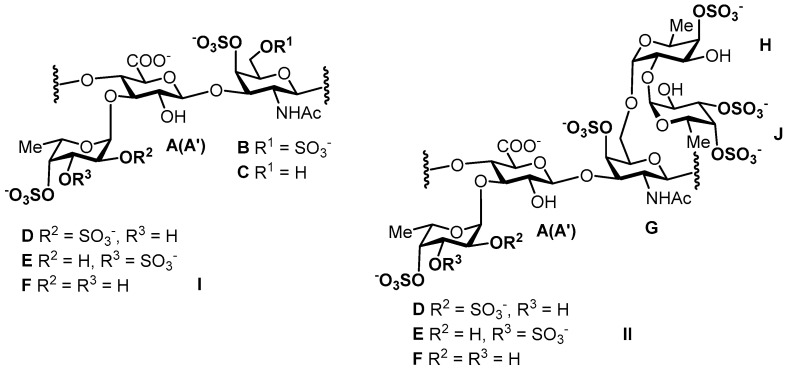
Repeating blocks of fucosylated chondroitin sulfates **PC** (units **A**–**D, F**) and **HH** (units **A**–**J**). Unit **A** bears Fuc2*S*4*S* (**D**), whereas unit **A’** bears Fuc3*S*4*S* (**E**) or Fuc4*S* (**F**).

**Figure 4:**
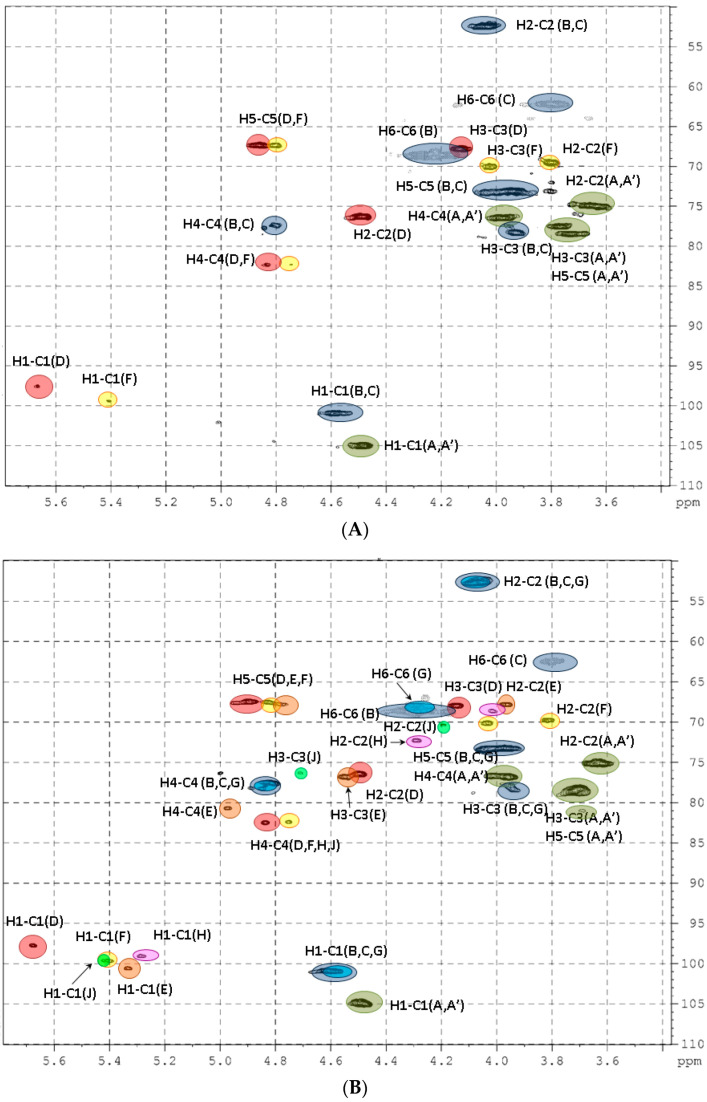
The HSQC NMR spectra of polysaccharides **PC** (**A**) and **HH** (**B**).

The application of 2D NMR experiments correlation spectroscopy (COSY), total correlation spectroscopy (TOCSY), heteronuclear single quantum coherence (HSQC), and rotating-frame nuclear Overhauser effect spectroscopy (ROESY) led to assigning all of the signals of the major components in the ^1^H and ^13^C NMR spectra of the polysaccharides (Figure 3 and Figure 4 and Figures S1–S3, Table 2). Analysis of the spectra of **PC** revealed the similarity of its structure to those described previously for FCS from other species of sea cucumbers [4,19,29]. Thus, the signals of GlcA, GalNAc, and Fuc units were related to the core [→3)-β-d-GalNAc-(1→4)-β-d-GlcA-(1→]_n_, bearing fucosyl branches at O-3 of every GlcA unit (see repeating unit **I** in Figure 3) [4,19]. There were two fucosyl units Fuc2*S*4*S* (**D**) and Fuc4*S* (**F**) that differed in pattern of sulfation, which was indicated by the downfield chemical shifts of the signals of respective protons in the ^1^H NMR spectrum (Table 2). The ratio of units **D** and **F** was determined using the integral intensities of the respective H-1 signals and was found to be 2:1. The linkages between the fucosyl units and O-3 of GlcA were confirmed by the correlation H-1(Fuc)-H-3(GlcA) in the ROESY spectrum (Figure S2). Sulfated GalNAc4*S*6*S* (**B**) and GalNAc4*S* (**C**) units were found in an approximate ratio of 3:2 in **PC** by integration of the intensities of the cross-peaks related to H-6–C-6 interaction in units **B** and **C** in the HSQC spectrum.

The structure of polysaccharide **HH** was more complex than that of **PC**. Two branched repeating blocks **I** and **II** were determined in **HH** (Figure 3). The first one was typical for all FCSs and contained three different fucosyl branches Fuc2*S*4*S* (**D**), Fuc3*S*4*S* (**E**), and Fuc4*S* (**F**) in a ratio of ~1.5:1:1 (calculated using the integral intensities of the respective H-1 signals, Figure S4). Units GalNAc4*S*6*S* (**B**) and GalNAc4*S* (**C**) were found in a ratio of 2:1. The repeating block **II** along with the fucosyl residue at O-3 of GlcA contained the unusual difucosyl branch attached to O-6 of GalNAc(**G**) and formed by units **H** and **J**. The chemical shift of the H-1 signal of unit **H** (*δ* 5.28 ppm) differed from those of units **D**, **E**, and **F**. This led to the assessment of the signals of the spin system of unit **H** using the COSY, TOCSY, and ROESY experiments (Figures S1–S3) and allowed for the determination of the signals of the respective carbon atoms from the HSQC spectrum (Figure 4B). The attachment of unit **H** to O-6 of GalNAc (**G**) was confirmed by the presence of the cross-peak H1(**H**)-H6(**G**) in the ROESY spectrum. The downfield chemical shift of the C-2 signal of **H** (*δ* 72.8 ppm) indicated the position of glycosylation (compared with *δ* 69.8 ppm for Fuc4*S* **F**). Detailed analysis of the ROESY spectrum revealed one more fucosyl unit **J** (*δ* 5.41 ppm) linked to O-2 of residue **H**, as the cross-peak H1(**J**)-H2(**H**) was detected. The positions of sulfate groups in units **H** and **J** were determined by the downfield chemical shifts of signals of the respective protons. Therefore, the presence of the unusual branch Fuc4*S*-(1→2)-Fuc3*S*4*S*-(1→ linked to O-6 of GalNAc was confirmed. The ratio of units **H** and **E** was estimated to be about 1:1 (Figure S4). Previously, difucosyl branches in holothurian FCSs were found to be attached to O-3 of GlcA but not to O-6 of GalNAc [8,9,10].

**Figure 5:**
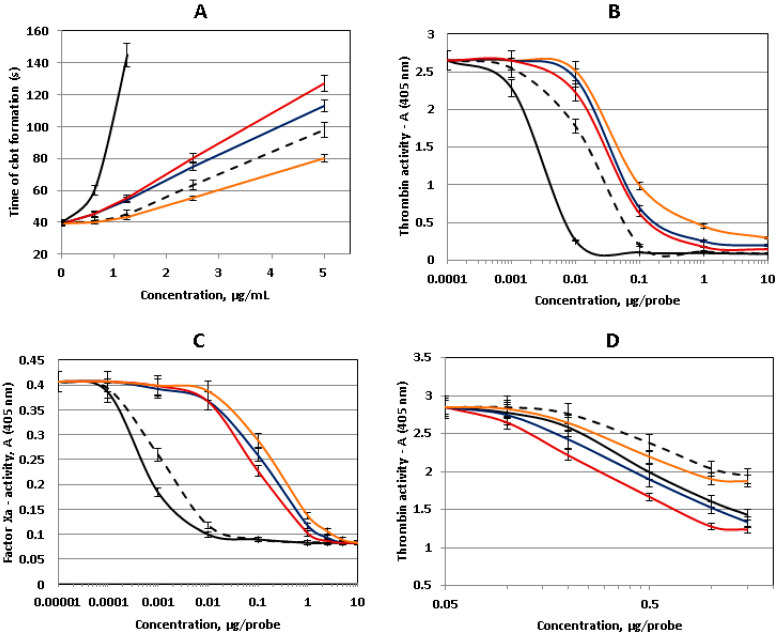
Anticoagulant activity of polysaccharides **PC** (blue), **HH** (red), **CD** (orange), heparin (black), and enoxaparin (dotted line). (**A**) Activate partial thromboplastin time (APTT) assay, (**B**) anti-IIa-activity in the presence of antithrombin III (ATIII), (**C**) anti-Xa-activity in the presence of ATIII, and (**D**) anti-IIa-activity without ATIII. *n* = 4, *p* < 0.05.

FCSs are known to demonstrate anticoagulant activity; therefore, we have studied two new polysaccharides **PC** and **HH** as anticoagulant agents in vitro. Heparin and low-molecular-weight heparin (enoxaparin) were used as standards. In addition, we have studied FCS **CD,** isolated previously from the sea cucumber *Cucumaria djakonovi* [30]. The latter polysaccharide includes the linear non-fucosylated disaccharide fragments →3)-β-d-GalNAc4*S*6*S*-(1→4)-β-d-GlcA-(1→, →3)-β-d-GalNAc4*S*-(1→4)-β-d-GlcA-(1→, and →3)-β-d-GalNAc6*S*-(1→4)-β-d-GlcA-(1→ along with the branched unit **I** (Figure 3). In the clotting time assay (activated partial thromboplastin time (APTT) test), the effects of **PC** and **HH** were higher than that of enoxaparin but lower than that of heparin, whereas polysaccharide **CD** was less active than enoxaparin (Figure 5A). The values of 2APTT (the concentration that led to a 2-fold increase of time of clot formation) were 0.8 ± 0.1 μg/mL for heparin, 2.5 ± 0.1 μg/mL for **HH**, 2.8 ± 0.1 μg/mL for **PC**, 3.7 ± 0.2 μg/mL for enoxaparin, and 5.0 ± 0.1 μg/mL for **CD**.

Thrombin and factor Xa are considered to be the main players in the coagulation cascade [1,6]. These serine proteases could be inhibited by ATIII, and this interaction is significantly increased in the presence of heparinoids. Therefore, we then studied the ability of the polysaccharides to potentiate the inhibition of thrombin and factor Xa in the presence of ATIII. In these experiments, all of the studied polysaccharides demonstrated the activity, but the values of the effects were lower than those of heparinoids (Figure 5B,C). Interesting results were obtained in the experiment with thrombin but without ATIII (Figure 5D). **HH** inhibited thrombin activity more effectively than **PC** and **CD**, and this phenomenon may be explained by the presence of disaccharide branches in **HH**. Notably, the activity of **HH** and **PC** was higher than that of heparin in this experiment. Previously, direct thrombin inhibition was described for fucoidans from brown seaweeds, the polysaccharides enriched in fucose content [31]. This mechanism might be taken into consideration, as it could impact the coagulation cascade.

## 3. Materials and Methods

### 3.1. General Methods

The procedures for the determination of neutral monosaccharides, sulfate, and uronic acids were described previously [32,33,34]. The molecular weights of polysaccharides were evaluated by chromatographic comparison with standard pullulans [28].

### 3.2. Isolation of Polysaccharides

The samples of sea cucumber *Holothuria hilla* were collected in the summer of 1990, on the seashore of D’Arros Island (Seyshelles) at a depth of 10 m by scuba divers. The taxonomic identification was made by Prof. V.S. Levin of the G.B. Elyakov Pacific Institute of Bioorganic Chemistry of the Far Eastern Branch of the Russian Academy of Sciences (PIBOC FEB RAS). The samples of *Paracaudina chilensis* were collected in the Trinity Bay, Peter the Great Gulf, the Sea of Japan in the summer of 2019 at a depth of 5–7 m. The taxonomic identification was carried out by Boris B. Grebnev (PIBOC FEB RAS). Both animals were fixed with ethanol. The sea cucumbers were cut into pieces, extracted twice with refluxed 70% EtOH, and the residue of animal material was air-dried.

According to the conventional procedure [27], dried and minced biomass of *H. hilla* (40 g) was suspended in 300 mL of 0.1 M sodium acetate buffer (pH 6.0), containing papain (1 g), ethylenediaminetetraacetic acid (EDTA) (0.4 g), and l-cysteine hydrochloride (0.2 g), and incubated at 45–50 °C for 24 h. After centrifugation, an aqueous hexadecyl-trimethylammonium bromide solution (10%, 30 mL) was added to the supernatant; the resulting precipitate was isolated by centrifugation and washed successively with water and ethanol. Then, it was stirred with a 20% ethanolic NaI solution (5 × 40 mL) for 2–3 days, washed with ethanol, dissolved in water, and lyophilized to obtain the crude polysaccharide preparation **HH-SP**, yield 1.9 g (4.7%), composition: Fuc 16.6%, uronic acids 3.0%, GlcN 2.1%, GalN 5.2%, Gal 2.1%, and SO_3_Na 15.8%. An aqueous solution of **HH-SP** (249 mg in 50 mL) was placed on a column (3 × 10 cm) with DEAE (Diethylaminoethyl)-Sephacel in Cl¯ form and eluted with water, followed by a NaCl solution of increasing concentration (0.5, 0.75, 1.0, and 1.5 M), each time until the absence of a positive reaction of eluate for carbohydrates [35]. Fractions were desalted on a Sephadex G-15 (Sigma-Aldrich, St. Louis, MO, USA. Catalog number: G15120) column and lyophilized. According to composition (Table 1), the fraction eluted with 1.0 M NaCl was designated as **HH** and studied further as preparation of FCS. Similar treatment of *P. chilensis* biomass (45 g) gave rise to crude polysaccharide preparation **PC-SP**, yield 1.2 g (2.7%), and preparation of FCS eluted from the DEAE-Sephadex with 1.0 M NaCl was designated as **PC** (Table 1).

### 3.3. NMR Spectroscopy

The NMR spectra were recorded using Zelinsky Institute Shared Research Facilities Center. The sample preparation and the conditions of the experiments were described previously [7].

### 3.4. Clotting Time Assay

The APTT test was performed as described previously [14]. Heparin (Sigma-Aldrich, St. Louis, MO, USA. Catalog number: 51550), enoxaparin (Clexane^®^, Sanofi, Paris, France), and FCS **CD** were used as references.

### 3.5. Effect of Polysaccharides on Thrombin or Factor Xa Inactivation by Antithrombin III

Both experiments were carried out at 37 °C in 96-well plates using MultiscanGo (Thermo Fisher Scientific, Stockholm, Sweden). Tris-HCl buffer was used as a control.

A ReaChrom ATIII test kit (Renam, Moscow, Russia) was used for the measurement of thrombin activity. A solution of a polysaccharide sample (**PC**, **HH**, **CD**, heparin, or enoxaparin) (20 µL) with concentrations of 500, 50, 5, 0.5, and 0.05 μg/mL in Tris-HCl buffer was added to 50 µL of a solution of ATIII (0.2 U/mL) in Tris-HCl buffer (0.15 µM, pH 8.4). After a 3-min incubation, an aqueous solution of thrombin (50 µL, 20 U/mL) was added, and the mixture was incubated for 2 min. Then, a chromogenic substrate (50 µL, 2 mM) was added, and the mixture was kept for 2 min. Absorbance of *p*-nitroaniline (405 nm) was measured.

A ReaChrom Heparin kit (Renam, Moscow, Russia) was used for the measurement of factor Xa activity. A solution of a polysaccharide sample (**PC**, **HH**, **CD**, heparin, or enoxaparin) (20 µL) with concentrations of 500, 50, 5, 0.5, and 0.05 μg/mL in Tris-HCl buffer was added to 50 µL of a solution of ATIII (0.5 U/mL) in Tris-HCl buffer. After a 3-min incubation, an aqueous solution of factor Xa (50 µL, 2 U/mL) was added, and the mixture was incubated for 2 min. The mixture was worked up, treated with chromogenic substrate, and analyzed as described above.

### 3.6. Effect of Polysaccharides on Thrombin Inactivation without ATIII

A ReaChrom ATIII test kit (Renam, Moscow, Russia) was used for the experiments. An aqueous solution of thrombin (50 µL, 20 U/mL) and a solution of a polysaccharide sample (**PC**, **HH**, **CD**, heparin, or enoxaparin) (20 µL) with concentrations of 750, 500, 250, 100, and 50 μg/mL in Tris-HCl buffer were added to 50 µL of Tris-HCl buffer. The mixture was incubated for 3 min. Then, 50 µL of a chromogenic substrate (2 mM) was added, and the incubation was continued for 2 min. The absorbance of *p*-nitroaniline (405 nm) was measured. Tris-HCl buffer was used as a control.

### 3.7. Statistical Analysis

All biological experiments were performed in quadruplicate (*n* = 4). The results are presented as Mean ± SD. Statistical significance was determined with Student’s t test. The *p* values less than 0.05 were considered significant.

## 4. Conclusions

Two new sulfated polysaccharides **PC** and **HH** were isolated from the sea cucumbers *Paracaudina chilensis* and *Holothuria hilla*, respectively. The main components of these biopolymers were GlcA, GalNAc, Fuc, and sulfate, indicating **PC** and **HH** as fucosylated chondroitin sulfates. Based on the data of the NMR spectra, both polysaccharides were shown to contain a chondroitin core [→3)-β-d-GalNAc-(1→4)-β-d-GlcA-(1→]_n_, bearing sulfated fucosyl branches at O-3 of every GlcA residue in the chain. These fucosyl residues were different in their pattern of sulfation: **PC** contained Fuc2*S*4*S* and Fuc4*S* in a ratio of 2:1, while **HH** included Fuc2*S*4*S*, Fuc3*S*4*S*, and Fuc4*S* in a ratio of 1.5:1:1. Moreover, some GalNAc residues in **HH** were found to contain the unusual disaccharide branch Fuc4*S*-(1→2)-Fuc3*S*4*S*-(1→ at O-6. Sulfated GalNAc4*S*6*S* and GalNAc4*S* units were found in a ratio of 3:2 in **PC** and 2:1 in **HH**. Both polysaccharides demonstrated significant anticoagulant activity in the clotting time assay, which is connected with the ability of these FCSs to potentiate inhibition of thrombin and factor Xa in the presence of ATIII and with the direct inhibition of thrombin in the absence of any cofactors.

## Figures and Tables

**Table 1:** Percentages of crude polysaccharide preparations and composition of fucosylated chondroitin sulfates **PC** and **HH** (in *w/w* %).

Polysaccharide	Yield	Fuc	GlcA	SO_3_Na	GlcN	GalN	Gal
**HH**	16.1	10.8	13.3	21.2	-	10.7	1.6
**PC**	20.9	10.9	11.4	21.3	1.2	8.8	4.3

**Table 2:** Chemical shifts of the signals in the ^1^H and ^13^C NMR spectra of the fucosylated chondroitin sulfates **PC** and **HH** (the bold numerals indicate the positions of sulfate).

Residue	H1/C1	H2/C2	H3/C3	H4/C4	H5/C5	H6/C6
**A**→4)-β-d-Glc*p*A-(1→	4.48/ 105.0	3.64/ 75.0	3.72/ 77.8	3.96/ 76.6	3.70/ 78.1	- 176.0
**A’**→4)-β-d-Glc*p*A-(1→	4.48/ 105.0	3.60/ 75.0	3.68/ 80.7	4.00/ 76.6	3.71/ 78.1	- 176.0
**B**→3)-β-d-Gal*p*NAc4*S*6*S*-(1→	4.58/ 100.9	4.07/ 52.7	3.95/ 77.9	**4.81/** **77.2**	4.00/ 73.2	**4.33, 4.20** **68.5**
**C**→3)-β-d-Gal*p*NAc4*S*-(1→	4.58/ 100.9	4.07/ 52.7	3.95/ 77.9	**4.81/** **77.2**	4.02/ 76.2	3.81/ 62.3
**D** α-l-Fuc*p*2*S*4*S*-(1→	5.68/ 97.7	**4.47/** **76.6**	4.17/ 67.8	**4.86/** **82.5**	4.90/ 67.5	1.37/ 16.9
**E** α-l-Fuc*p*3*S*4*S*-(1→	5.34/ 100.5	3.95/ 67.6	**4.53/** **76.6**	**4.99/** **80.6**	4.80/ 67.6	1.37/ 17.2
**F** α-l-Fuc*p*4*S*-(1→	5.40/ 99.6	3.82/ 69.7	4.04/ 70.0	**4.77/** **82.4**	4.80/ 67.6	1.37/ 17.2
**G**→3)-β-d-Gal*p*NAc4*S*-(1→	4.47/ 105.1	3.38/ 73.9	3.59/ 75.2	**4.81/** **77.2**	4.02/ 76.2	3.81/ 62.3
**H**→2)-α-l-Fuc*p*4*S*-(1→	5.28/ 99.1	4.28/ 72.8	4.00/ 68.6	**4.84/** **82.5**	NdNd	1.37/ 17.2
**J** α-l-Fuc*p*3*S*4*S*-(1→	5.41/ 99.6	4.19/ 70.9	**4.69/** **76.6**	**4.99/** **80.6**	NdNd	1.37/ 17.2

Nd—not determined.

## Supplementary Materials

The following are available online at https://www.mdpi.com/1660-3397/18/11/540/s1, Figure S1: The COSY NMR spectra of polysaccharides **PC** (**I**) and **HH** (**II**), Figure S2: The ROESY NMR spectra of polysaccharides **PC** (**I**) and **HH** (**II**), Figure S3: The TOCSY NMR spectra of polysaccharides **PC** (**I**) and **HH** (**II**), and Figure S4: The calculation of the ratios of units **H:E** and **D:E:F** in polysaccharide **HH** using the integral intensities of the respective H-1 signals.
