# Supplementary material for: Fucosylated Chondroitin Sulfates from the Sea Cucumbers Paracaudina chilensis and Holothuria hilla: Structures and Anticoagulant Activity
Source: Mar Drugs. 2020 Oct 28;18(11):540. doi: 10.3390/md18110540 (PMC7693656; doi:10.3390/md18110540)
Supplement: Supplementary file 1 [file marinedrugs-18-00540-s001.pdf]

marine drugs

MDPI

Supplementary information

Fucosylated Chondroitin Sulfates from the Sea Cucumbers *Paracaudina Chilensis* and *Holothuria Hilla*: Structures and Anticoagulant Activity

Nadezhda E. Ustyuzhanina [1,*], Maria I. Bilan [1], Andrey S. Dmitrenok [1], Alexandra S. Silchenko [2], Boris B. Grebnev [2], Valentin A. Stonik [2], Nikolay E. Nifantiev [1], and Anatolii I. Usov [1,*]

[1] N.D. Zelinsky Institute of Organic Chemistry, Russian Academy of Sciences, Leninsky prospect 47, Moscow 119991, Russia; bilan@ioc.ac.ru (M.I.B.); dmt@ioc.ac.ru (A.S.D.); nen@ioc.ac.ru (N.E.N.)

[2] G.B.Elyakov Pacific Institute of Bioorganic Chemistry, Far Eastern Branch of the Russian Academy of Sciences, prospect 100 let Vladivostoku 159, Vladivostok 690022, Russia; sialexandra@mail.ru (A.S.S); grebnev_bor@mail.ru (B.B.G.); stonik@piboc.dvo.ru (V.A.S.)

* Correspondence: ustnad@gmail.com (N.E.U.); usov@ioc.ac.ru (A.I.U.); Tel.: +7-495-135-8784 (N.E.U.)

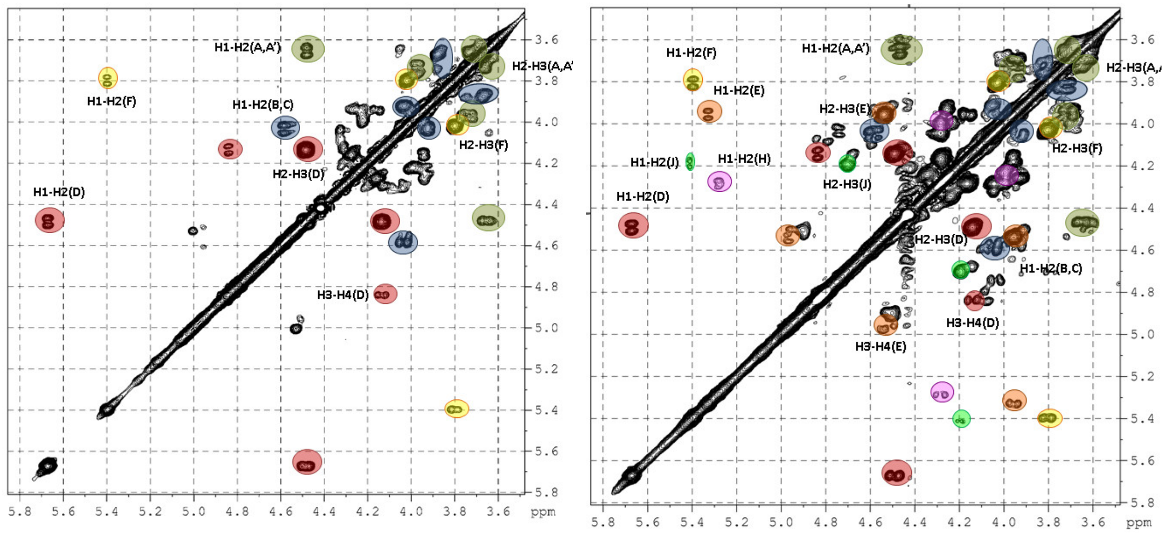

I **II**

Figure S1. The COSY NMR spectra of polysaccharides **PC** (**I**) and **HH** (**II**).

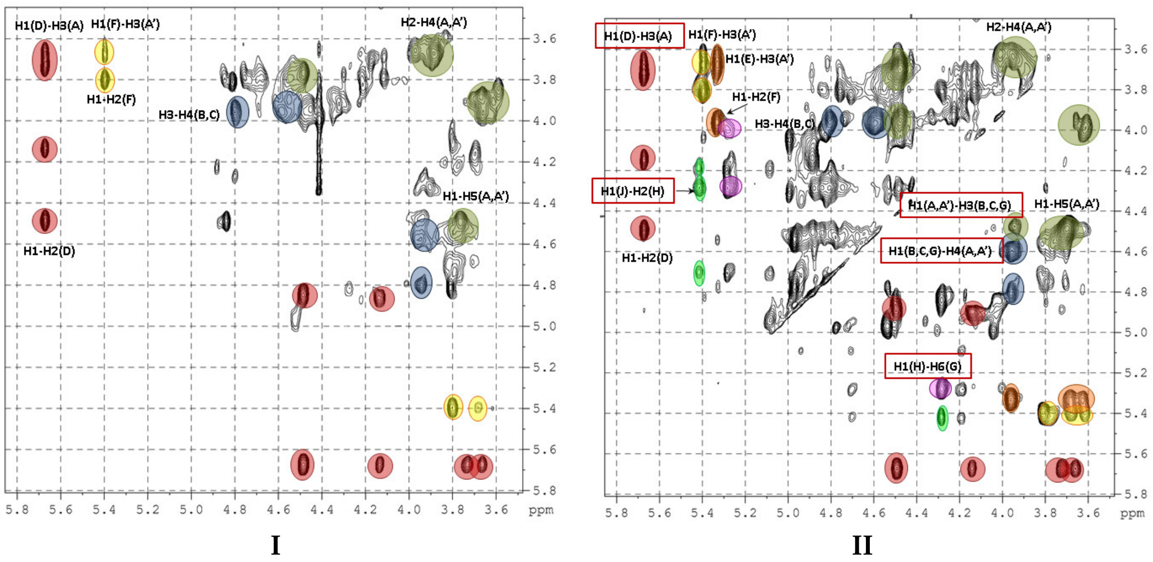

Figure S2. The ROESY NMR spectra of polysaccharides **PC** (**I**) and **HH** (**II**).

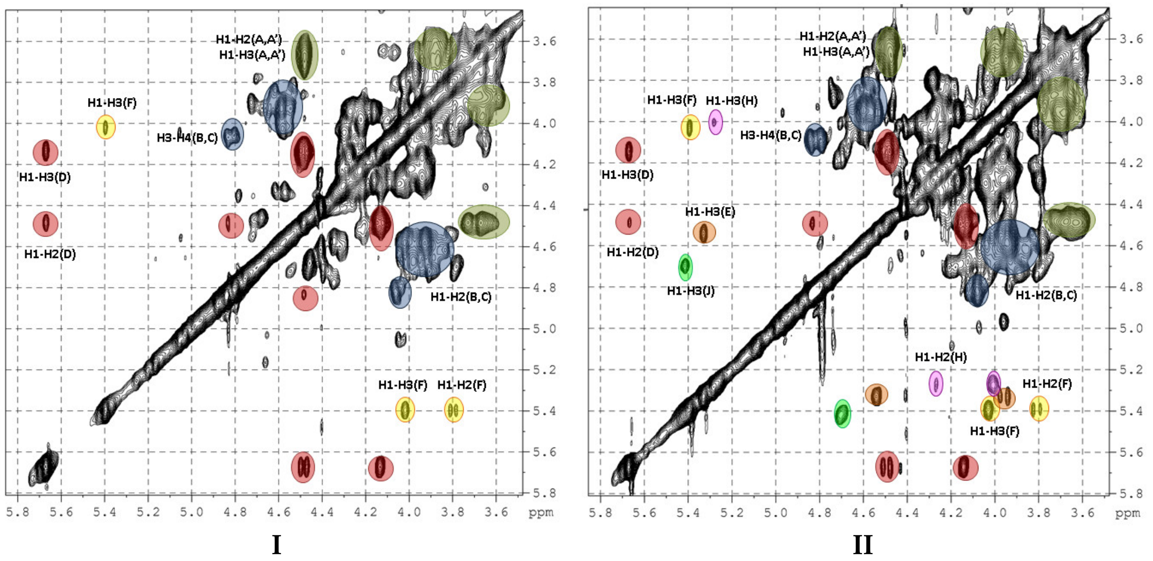

Figure S3. The TOCSY NMR spectra of polysaccharides **PC** (**I**) and **HH** (**II**).

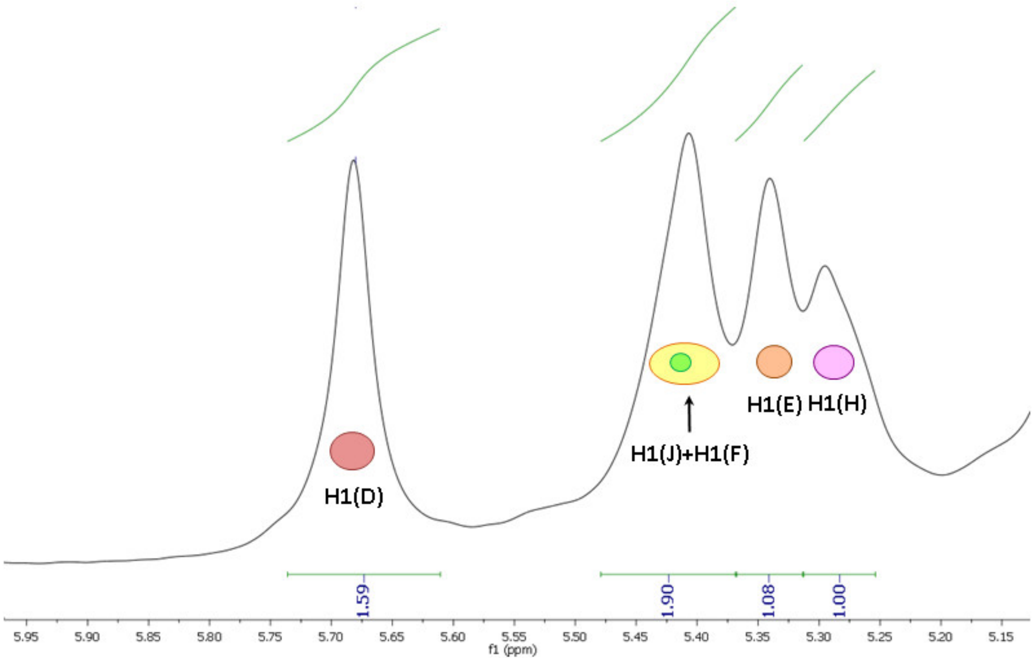

$$\int \boldsymbol{H1(J)} = \int \boldsymbol{H1(H)} = \mathbf{1}$$

$$\int \boldsymbol{H1(F)} = \mathbf{1.90} - \int \boldsymbol{H1(J)} = \mathbf{1.90} - \mathbf{1.00} = \mathbf{0.9}$$

⇒ the ratio of units H:E is ~ 1:1

the ratio of units D:E:F is ~ 1.5:1:1

Figure S4. Calculation of the ratios of units **H:E** and **D:E:F** in polysaccharide **HH** using the integral intensities of the respective H-1 signals.

© 2020 by the authors. Licensee MDPI, Basel, Switzerland. This article is an open access article distributed under the terms and conditions of the Creative Commons Attribution (CC BY) license (http://creativecommons.org/licenses/by/4.0/).
